# Differences in place of death between lung cancer and COPD patients: a 14-country study using death certificate data

**DOI:** 10.1038/s41533-017-0017-y

**Published:** 2017-03-03

**Authors:** Joachim Cohen, Kim Beernaert, Lieve Van den Block, Lucas Morin, Katherine Hunt, Guido Miccinesi, Marylou Cardenas-Turanzas, Bregje Onwuteaka-Philipsen, Rod MacLeod, Miguel Ruiz-Ramos, Donna M Wilson, Martin Loucka, Agnes Csikos, Yong-Joo Rhee, Joan Teno, Winne Ko, Luc Deliens, Dirk Houttekier

**Affiliations:** 1 Vrije Universiteit Brussel (VUB) and Ghent University, End-of-Life Care Research Group, Brussels, Belgium; 2 Department of Family Medicine and Chronic Care, Vrije Universiteit Brussel (VUB), Brussels, Belgium; 3 National Observatory of End of Life Care, Paris, France; 4 Aging Research Center, Karolinska Institutet and Stockholm University, Stockholm, Sweden; 5 University of Southampton, Health Sciences, Southampton, UK; 6 Cancer Prevention and Research Institute, ISPO, Clinical and Descriptive Epidemiology Unit, Florence, Italy; 7 The University of Texas MD Anderson Cancer Center, Houston, TX USA; 8 EMGO Institute for Health and Care Research, Public and Occupational Health, and Palliative Care Expertise Centre, VU Medical Centre, Amsterdam, The Netherlands; 9 Hammond Care and University of Sydney, Sydney, Australia; 10 Ministry of Health, Government of Andalusia, Seville, Spain; 11 Faculty of Nursing, University of Alberta, Edmonton, AB Canada; 12 Center for Palliative Care, Third Faculty of Medicine, Charles University in Prague, Prague, Czech Republic; 13 University of Pécs Medical School, Pécs, Hungary; 14 Dongduk Women’s University, Health Sciences, Seoul, Republic of Korea; 15 University of Washington, Cambia Palliative Care Center of Excellence, Division of Gerontology and Geriatrics, Seatle, WA USA; 16 Department of Medical Oncology, Ghent University, Ghent, Belgium

## Abstract

Chronic obstructive pulmonary disease and lung cancer are leading causes of death with comparable symptoms at the end of life. Cross-national comparisons of place of death, as an important outcome of terminal care, between people dying from chronic obstructive pulmonary disease and lung cancer have not been studied before. We collected population death certificate data from 14 countries (year: 2008), covering place of death, underlying cause of death, and demographic information. We included patients dying from lung cancer or chronic obstructive pulmonary disease and used descriptive statistics and multivariable logistic regressions to describe patterns in place of death. Of 5,568,827 deaths, 5.8% were from lung cancer and 4.4% from chronic obstructive pulmonary disease. Among lung cancer decedents, home deaths ranged from 12.5% in South Korea to 57.1% in Mexico, while hospital deaths ranged from 27.5% in New Zealand to 77.4% in France. In chronic obstructive pulmonary disease patients, the proportion dying at home ranged from 10.4% in Canada to 55.4% in Mexico, while hospital deaths ranged from 41.8% in Mexico to 78.9% in South Korea. Controlling for age, sex, and marital status, patients with chronic obstructive pulmonary disease were significantly less likely die at home rather than in hospital in nine countries. Our study found in almost all countries that those dying from chronic obstructive pulmonary disease as compared with those from lung cancer are less likely to die at home and at a palliative care institution and more likely to die in a hospital or a nursing home. This might be due to less predictable disease trajectories and prognosis of death in chronic obstructive pulmonary disease.

## Lung disease: improving end-of-life care

Structured palliative care similar to that offered to cancer sufferers should be in place for patients with chronic lung disease. Joachim Cohen at Vrije University in Brussels and co-workers examined international death certificate data collected from 14 countries to determine place of death for patients with lung cancer and chronic obstructive pulmonary disease (COPD). While patients with COPD suffer similar symptoms to lung cancer in their final days, few COPD patients receive palliative care or achieve the common wish of dying at home. This may be partly due to the inherent unpredictability of final-stage COPD compared with lung cancer. Cohen’s team found that, with the exception of Italy, Spain, and Mexico, patients with COPD were significantly more likely to die in hospital than at home. They highlight the need for improved COPD palliative care provision.

## Introduction

Lung cancer and chronic obstructive pulmonary disease (COPD) are two major causes of death in many countries, appearing as the fifth and third most common cause of death globally.^1^ Both illnesses affect patients’ quality of life with various stages of functional decline before death. Studies suggested that patients from both disease groups suffer from considerable dyspnea and pain.^2–4^ Other studies have indicated that people with COPD have severe symptoms causing major disruptions to normal life but these are often perceived and accepted as a “way of life” rather than an illness.^5, 6^ Despite similar problems, existing literature has reported a disadvantage for people with COPD compared with those with lung cancer in receiving end-of-life care.^3, 4, 7, 8^ Lung cancer patients seem to receive a more holistic palliative approach to care.^3^ Fewer palliative care resources were used by people with COPD^3, 4, 9, 10^ and end-of-life care discussions occurred later in their disease trajectories.^11^ Those with COPD also seem to face unmet care needs to a larger extent^12, 13^ and appear to have less access to palliative care services.^9, 14^ The historical focus of palliative care on cancer patients may be one reason for this.^7^ Another reason might be the difference in disease trajectories.^15^ Predicting outcomes or prognosis is often easier for patients with lung cancer with a rapid decline and a distinct terminal phase than for those with COPD who have more acute exacerbations and have less recognizable terminal phase.^16^

Previous research comparing end-of-life care for COPD and lung cancer patients has focused on symptom management^17–19^ and communication,^14, 20, 21^ little is known about how place of death differs between them. Place of death is often seen as a contributing factor in quality of dying, particularly because most people prefer to be cared for and to die at home.^22, 23^ The setting of dying has been shown to influence the characteristics of care and the dying experience. From research using Medicare data from the United States (USA), we know that COPD patients were more likely to die in hospital than were lung cancer patients.^24^ Nonetheless, cross-national comparisons for both populations remain scarce and such studies encourage mutual learning across borders by shedding a light on how patients with the same or different diseases die in different countries. Even neighboring countries with relatively similar cultures may organize end-of-life care differently and this evidence is valuable for evidence-based health policy-making.

The research aims of this study were first to compare and describe place of death of those persons diagnosed with lung cancer compared with those diagnosed with COPD in 14 countries and second to examine to what extent place of death differences between the two disease groups are due to confounding socio-economic and residential factors.

## Results

### Country and death characteristics

A total of 5,568,827 deaths were documented in the 14 countries. In all countries, except New Zealand and Mexico, more people died from lung cancer than COPD (Table 1, country abbreviations explained). Lung cancer deaths ranged from 1.2% of all in Mexico to 7.6% in the Netherlands. Deaths from COPD ranged from 1.7% in France to 5.3% in the USA.

**Table 1:** Deaths from COPD and lung cancer in 14 countries during the year 2008 (*N* = 5,568,827)

Country	Abbreviations	Total number of deaths	COPD deaths (*N*)	COPD deaths (% of all deaths)	Lung cancer deaths (*N*)	Lung cancer deaths (% of all deaths)
France	FR	541,135	9274	1.7	29,221	5.4
Italy	IT	578,192	21,356	3.7	33,004	5.7
Spain (Andalusia)	ES	57,380	2564	4.5	3198	5.6
Belgium	BE	102,924	4751	4.6	6491	6.3
The Netherlands	NL	135,136	6303	4.9	9918	7.6
The Czech Republic	CZ	101,804	2161	2.1	5310	5.2
Hungary	HU	130,027	4875	3.7	8330	6.4
England	ENG	475,763	25,143	5.3	28,222	5.9
Wales	WAL	32,066	1730	5.4	2032	6.3
New Zealand	NZ	29,312	1837	6.3	1634	6.0
Canada	CA	182,134	8185	4.5	12,902	7.1
United States of America	USA	2,428,343	12,8021	5.3	15,8889	6.5
Mexico	MX	528,093	21,804	4.6	6563	1.2
Korea	KR	247,757	7349	3.0	14,883	6.0
Total		5,568,827	245,345	4.4	320,591	5.8

*COPD* chronic obstructive pulmonary disease

As compared with people dying from lung cancer, those dying from COPD were more often older, female, and widowed or divorced (Table 2). Most lung cancer patients were married (England: 51.3% to Italy: 69.3%), whereas those with majority COPD were more often widowed or divorced (Spain: 37.6% to USA: 57.5%).

**Table 2:** Demographic characteristics of people with COPD or lung cancer who died during 2008 in 14 countries (*N* = 562,151)

		Number of deaths	Age (%)			Sex (%)	Marital status (%)		
			18–64	65–84	85 or older	Female	Unmarried	Married	Widowed/divorced
France	COPD	9274	10.2	30.0	59.6	37.5	12.4	40.4	47.3
	Lung cancer	29,221	40.4	40.3	19.3	23.6	11.4	60.8	27.8
Italy	COPD	21,356	3.5	26.2	70.3	39.3	10.8	42.5	46.7
	Lung cancer	33,004	22.3	50.7	27.0	23.4	8.1	69.3	22.6
Spain (Andalusia)	COPD	2564	6.0	38.7	55.2	20.6	9.1	53.3	37.6
	Lung cancer	3198	33.1	47.1	19.8	13.7	8.5	71.6	19.9
Belgium	COPD	4751	11.5	37.2	51.2	36.5	8.2	43.0	48.8
	Lung cancer	6491	30.9	47.3	21.8	23.8	7.1	61.3	31.7
The Netherlands	COPD	6303	9.4	36.8	53.7	44.2	59.7	40.3	N/A
	Lung cancer	9918	31.5	48.5	20.0	35.6	38.9	61.1	N/A
The Czech Republic	COPD	2161	21.4	42.4	36.2	39.7	8.0	39.9	52.1
	Lung cancer	531	39.6	46.7	13.6	27.4	5.7	57.2	37.1
Hungary	COPD	4875	15.8^a^	48.4^a^	35.7^a^	40.9	9.5	36.3	54.1
	Lung cancer	833	33.5^a^	55.5^a^	10.9^a^	32.8	7.5	53.6	38.9
England	COPD	25,143	10.5	39.2	50.1	48.7	7.8	36.9	55.3
	Lung cancer	28,222	22.3	48.2	29.5	43.2	6.8	51.3	41.9
Wales	COPD	173	9.1	40.8	50.1	50.8	6.0	39.0	54.9
	Lung cancer	2032	22.5	49.2	28.3	42.4	6.1	51.8	42.1
New Zealand	COPD	1837	12.2	37.8	49.9	50.2	^b^	^b^	^b^
	Lung cancer	1634	28.8	47.1	24.1	45.6	^b^	^b^	^b^
Canada	COPD	8185	8.5	34.3	57.1	46.9	7.7	36.8	55.6
	Lung cancer	12,902	26.7	47.7	25.7	46.0	6.8	55.5	37.7
USA	COPD	128,021	14.2	40.2	45.4	52.1	6.3	36.2	57.5
	Lung cancer	158,889	28.8	47.1	24.1	44.3	6.0	51.0	43.0
Mexico	COPD	21,804	11.8	35.9	51.4	44.5	11.2	44.6	44.2
	Lung cancer	6563	31.3	47.3	21.3	33.7	10.8	61.8	27.4
Korea	COPD	7349	7.9	42.8	49.3	38.7	2.8	47.7	49.7
	Lung cancer	14,883	25.3	56.3	18.4	26.4	2.3	69.2	28.5

*Note*: Percentages are row percentages. Percentages may not add up to 100 due to rounding

^a^For age, the Hungarian file was delivered using a different aggregation: 0–17, 18–59, 60–79 (65–84), 80 or above (85 or above)

^b^Variable not available for the country; N/A: category within variable not presented on the data file of the Netherlands; variable was dichotomized into married or not

### Place of death

From 12.5% (Korea) to 57.1% (Mexico) of persons diagnosed with lung cancer died at home (Table 3). Hospital deaths accounted for 27.5% (New Zealand) to 86.5% (Korea) of lung cancer deaths. Another 0.9% (Korea) to 22.5% (New Zealand) of lung cancer deaths occurred in nursing homes. For countries where the category hospice (i.e., palliative care institution) was available (England, Wales, New Zealand, and the USA), from 5.2% (USA) to 17.6% (New Zealand) of lung cancer deaths took place there. Of the COPD deaths, 10.4% (Canada) to 55.4% (Mexico) took place at home, 41.8% (The Netherlands) to 78.9% (Korea) in hospital, 1.5% (Korea) to 35.4% (The Netherlands) in nursing homes, and 0.2% (Wales) to 2.9% (USA) in palliative care institutions. A comparison with the place of death distribution for all-cause mortality can be found in the supplementary online appendix (Table S1).

**Table 3:** The place of death of deceased patients with COPD and lung cancer by country (*N* = 562,151)

		Number of deaths	Home (%)	Hospital (%)	Nursing home (%)	Hospice setting (%)	Others (%)
France	COPD	9274	25.6	60.2	11.2	/^a^	3.0
	Lung cancer	29,221	17.0	77.4	3.0	/^a^	2.7
Italy	COPD	21,356	41.6	47.4	7.5	/^a^	3.5
	Lung cancer	33,004	44.2	49.5	2.9	/^a^	3.5
Spain (Andalusia)	COPD	2564	36.3	56.7	6.2	/^a^	.8
	Lung cancer	3198	33.1	64.5	2.1	/^a^	.2
Belgium	COPD	4751	22.0	52.8	24.4	/^a^	.8
	Lung cancer	6491	28.8	63.9	6.8	/^a^	.6
The Netherlands	COPD	6303	20.5	41.8	35.4	1.5^b^	0.8
	Lung cancer	9918	48.0	28.0	15.3	7.2^b^	1.5
The Czech Republic	COPD	2161	17.2	66.8	14.7	/^a^	1.3
	Lung cancer	531	17.3	66.4	15.5	/^a^	.8
Hungary	COPD	4875	/^a^	69.8	/^a^	/^a^	30.2
	Lung cancer	833	/^a^	72.1	/^a^	/^a^	27.9
England	COPD	25,143	19.8	67.5	10.8	.9	1.0
	Lung cancer	28,222	28.2	45.8	9.0	15.3	1.7
Wales	COPD	173	16.9	73.9	8.2	.2	.8
	Lung cancer	2032	28.1	57.5	4.2	8.5	1.6
New Zealand	COPD	1837	15.5	44.7	34.0	1.9	4.0
	Lung cancer	1634	29.9	27.5	22.5	17.6	2.6
Canada	COPD	8185	10.4	65.4	20.7	/^a^	3.6
	Lung cancer	12,902	16.3	69.0	9.9	/^a^	4.9
USA	COPD	128,021	26.2	44.5	22.7	2.9	3.7
	Lung cancer	158,889	40.3	33.9	15.0	5.2	5.7
Mexico	COPD	21,804	55.4	41.8	/^a^	/^a^	2.8
	Lung cancer	6563	57.1	40.1	/^a^	/^a^	2.8
Korea	COPD	7349	19.4	78.9	1.5	/^a^	.3
	Lung cancer	14,883	12.5	86.5	.9	/^a^	.1

*Note*: Percentages are row percentages. Percentages may not add up to 100 due to rounding

^a^Category not presented on death certificate. In Hungary, the death certificate registry only coded hospital or others as the place of death and nursing home does not exist as a separate health service in Mexico

^b^In the Netherlands, the category “other institutions” on the death certificate mostly concerns hospices in these disease groups

### Comparison of the place of death for COPD and lung cancer sufferers

**Fig. 1:**
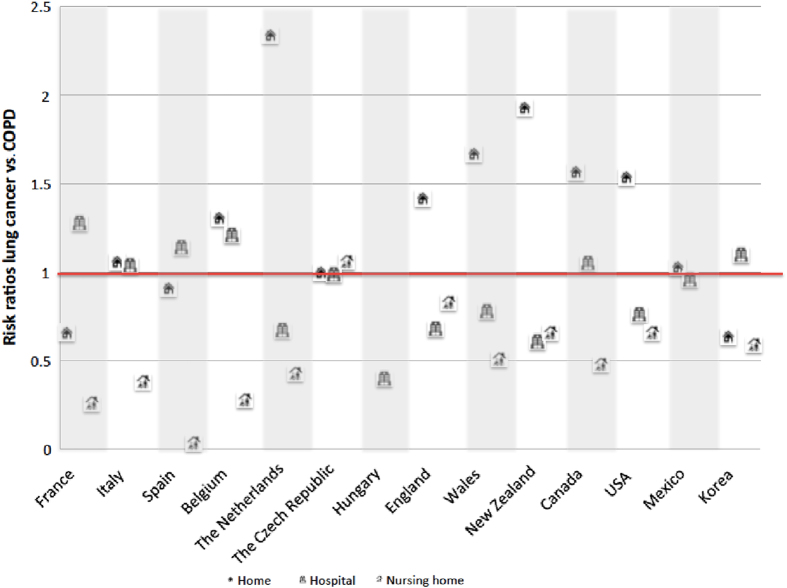
Crude risk ratios for dying at home, in hospital, in a nursing home, in a palliative care institution of those dying from lung cancer vs. those from COPD. Risk ratios calculated as proportion lung cancer/proportion COPD dying in each place. A risk ratio >1 indicates that those with lung cancer were relatively more likely to die in that place compared to those with COPD deaths; a risk ratio <1 means that they were less likely

As compared with COPD sufferers, those with lung cancer had higher crude chances of dying at home in nine countries, with the difference particularly large in the Netherlands (2.34) and New Zealand (1.93) (Fig. 1). In six of these nine countries, lung cancer patients were less likely to die in hospitals. In three countries—Belgium, Italy, and Canada—persons with lung cancer had both a higher ratio of dying at home and in hospital compared with people with COPD. Those with lung cancer in all countries (except in the Czech Republic) were less likely to die in a nursing home. In countries where palliative care institutions were available as a category of place of death, lung cancer sufferers were more likely than COPD ones to die there [risk ratios: 17.93 (England), 36.82 (Wales), 9.52 (New Zealand), and 1.78 (USA)] (not presented in figure).

### Multivariable analyses

#### Home vs. any other place of death

Controlling for confounders (age, sex, marital status), using binary logistic regression analyses (Table 4), persons dying of COPD were significantly less likely than lung cancer patients to die at home in 10 countries [OR from 0.4 (The Netherlands) to 0.8 (Belgium, Spain, and Mexico)]. An opposite pattern was found in France (OR 1.7) and Korea (OR 1.5), with COPD sufferers there more likely to die at home.

**Table 4:** Odds ratios of COPD vs. lung cancer patients (reference) stratified by place of death: binary multivariable logistic regression models of death certificates data from 14 countries during 2008 (*N* = 562,151)

Country	Home (vs. all others) (*N* = 562,151)	Home (vs. hospital) (*N* = 447,537)	Hospital (vs. all others) (*N* = 562,151)	Nursing home (vs. hospital) (*N* = 345,463)	Hospice setting (vs. others) (*N* = 41,092)
	OR (95% CI)	OR (95% CI)	OR (95% CI)	OR (95% CI)	OR (95% CI)
France	**1.7 (1.58**–**1.80)**	**1.8 (1.71**–**1.94)**	**0.5 (0.52**–**0.58)**	**2.1 (1.89**–**2.33)**	/^a^
Italy	**0.9 (0.87**–**0.94)**	**0.9 (0.88**–**0.96)**	**1.0 (1.00**–**1.08)**	**1.6 (1.42**–**1.72)**	/^a^
Spain	**0.8 (0.75**–**0.96)**	0.9 (0.79–1.01)	1.0 (0.91–1.17)	**1.9 (1.34**–**2.67)**	/^a^
Belgium	**0.8 (0.77**–**0.93)**	0.97 (0.9–1.07)	**0.8 (0.71**–**0.84)**	**2.4 (2.09**–**2.73)**	/^a^
The Netherlands	**0.4 (0.33**–**0.39)**	**0.3 (0.27**–**0.33)**	**2.4 (2.23**–**2.59)**	**0.9 (0.81**–**0.98)**	/^a^
The Czech Republic	1.1 (0.96–1.26)	1.1 (0.92–1.22)	1.1 (0.95–1.19)	**0.8 (0.68**–**0.92)**	/^a^
Hungary	/^a^	/^a^	0.9 (0.85–1.01)	/^a^	/^a^
England	**0.7 (0.68**–**0.74)**	**0.5 (0.50**–**0.54)**	**2.5 (2.42**–**2.60)**	**0.6 (0.55**–**0.62)**	**0.1 (0.07**–**0.11)**
Wales	**0.6 (0.49**–**0.68)**	**0.5 (0.43**–**0.61)**	**2.1 (1.80**–**2.40)**	1.1 (0.82–1.48)	**0.1 (0.01**–**0.20)**
New Zealand	**0.5 (0.43**–**0.60)**	**0.3 (0.27**–**0.40)**	**2.7 (2.28**–**3.10)**	**0.6 (0.51**–**0.75)**	**0.1 (0.05**–**0.14)**
USA	**0.6 (0.56**–**0.58)**	**0.5 (0.47**–**0.49)**	**1.8 (1.81**–**1.87)**	**0.8 (0.77**–**0.81)**	**0.9 (0.87**–**0.98)**
Canada	**0.7 (0.63**–**0.76)**	**0.7 (0.67**–**0.80)**	0.98 (0.92–1.05)	**1.5 (1.40**–**1.66)**	/^a^
Mexico	**0.8 (0.72**–**0.82)**	**0.8 (0.72**–**0.81)**	**1.3 (1.21**–**1.37)**	/^a^	/^a^
Korea	**1.5 (1.43**–**1.68)**	**1.6 (1.43**–**1.69)**	**0.7 (0.60**–**0.71)**	1.2 (0.90–1.57)	/^a^

*Note*: Bold denotes a significant difference between lung cancer and COPD patients. Variables included in model: age, sex, marital status (except NZ, where marital status was not available)

^a^Category did not exist on death certificate

#### Home vs. hospital as place of death

Lower odds ratios for home death (less than one) were observed in COPD decedents in nine countries [OR from 0.3 (The Netherlands and New Zealand) to 0.9 (Spain)] with France (OR 1.8) and Korea (OR 1.6) showing patients with COPD more likely to die in hospitals.

#### Hospital vs. any other place of death

Compared with those with lung cancer, COPD patients were significantly more likely to die in hospital instead of outside a hospital in seven countries [OR from 1.0 (Italy) to 2.7 (New Zealand)], but the opposite was observed in France (OR 0.5), Korea (OR 0.7), and Belgium (OR 0.8). The Czech Republic was the only country showing no differences between the two disease groups with regard to place of death.

#### Nursing home vs. hospital as place of death

COPD patients were more likely than lung cancer patients to die in a nursing home in five countries [OR from 1.5 (Canada) to 2.4 (Belgium)], while in five other countries (The Netherlands, The Czech Republic, England, New Zealand, USA) COPD decedents died more often in hospitals than did lung cancer decedents.

#### Palliative care institutions vs. any other place of death

Lastly, a comparison between palliative care institutions and other places of death for England, Wales, New Zealand, and the USA showed that in all these countries COPD decedents had a significantly lower chance of dying in a palliative care institution compared with lung cancer decedents (ORs ranging from 0.1 to 0.9).

## Discussion

### Main findings

This study captures variations in place of death of people dying from lung cancer and COPD, two major causes of death, across different health-care systems. We found that patients dying from COPD were more likely to die in hospital than at home (or in a palliative care institution) than those dying from lung cancer, even when considering characteristics in terms of age, gender, and marital status. France and Korea were the exceptions.

### Strengths and limitations of this study

Using population death certificate data to study cross-national variation in place of death may induce some limitations that need to be taken into account.^25^ Due to coding and processing delays in some countries and our stipulation to select the same year across the different countries the data used are relatively old (2008) and it may be that some changes in the place of death have occurred in some countries since then.^26^ However, no other previous study has examined cross-national variation in place of death of people who died from lung cancer or COPD in both European and non-European countries. Death certificates have been shown to sometimes be inaccurate in recording the correct cause of death.^27, 28^ COPD is known to be under-reported on death certificates.^29^ However, for the purpose of comparison with those who died from lung cancer (which may be less prone to underreporting as a cause of death) and selection of only those people whose recorded underlying cause of death was COPD (i.e., those who died from COPD and thus were probably in an advanced stage and with a clear diagnosis of COPD at the time of death), this problem of under-reporting may be less problematic. An additional limitation, inherent to using robust population-level data, is that a loss of information at the individual level is inevitable. For instance, the death certificate does not provide information on important aspects of the end-of-life process such as preferences of place of death, choices of place of care, and course of decision-making levels, i.e., patients, family, health-care professionals, and/or health-care policy makers. Nevertheless, the statistical patterns about place of death do reflect important differences in the health-care organizational choices countries have made regarding end-of-life care in lung cancer vs. COPD and inspire further studies to provide us with a deeper understanding of observed patterns and the cross-national differences underlying those patterns.

### Interpretation of findings in relation to previously published work

Previous studies have found on average 75% of respondents prefer to die at home, among the terminally ill and the general public.^22^ However, for the majority of countries in our study, COPD decedents were substantially more likely than lung cancer decedents to die in hospital even after controlling for confounders; this may suggest a lack of options for COPD patients to die at home in most countries. This is likely to be due to a combination of factors, including a long-standing cancer focus on palliative care. COPD is an illness characterized by unpredictable exacerbations and prognosis of death. There is no clear-cut indicator with enough evidence to be effective to estimate the end-of-life phase for COPD.^30^ In addition, these patients and their family caregivers often do not realize that they have a limited life expectancy.^8, 31^ However, recently a cluster randomized controlled trial has shown that advance care planning in COPD sufferers might decrease hospitalizations and increases home deaths.^32^ Previous experiences have indicated that working with a coordinator for care planning may also be a way to improve end-of-life care for persons diagnosed with COPD.^33^ Making of advance care plans and establishing contact with end-stage care services could possibly result in a reduction of hospitalizations of advanced COPD patients at the end of life and increase the opportunity to honor their preferences for place of death.

We found that the percentages of COPD patients dying in nursing homes are substantially higher compared with lung cancer. This finding may reflect their older age and their disability or loss of functional performance in home management.^34^ This functional performance was found to be higher in older females,^34^ often leading to admission to a nursing home, and might explain why female COPD patients are more likely to die in a nursing home. However, the role of the nursing home as a place of end-of-life care and death is not well understood. Previous studies highlighted barriers to performing end-of-life care in long-term care settings because of a lack of communication and failure to initiate a palliative trajectory in good time.^35, 36^ This is an important consideration as hospital deaths can potentially be avoided if location preferences are known through optimal advance care planning. Improving the quality of end-of-life care in nursing homes, including policies to reduce hospitalizations at the end of life, thus seems to be an important policy priority for COPD sufferers as opposed to simply focusing all efforts on enabling them to die at home.

While the differences in terms of place of death between COPD and lung cancer decedents were large in most countries, they were very small in Italy, Spain, and Mexico. In these three countries, a relatively large proportion of both COPD and lung cancer patients died at home. This is probably due to a culture of family (or community) caregiving rather than the result of specific public health policies to facilitate home deaths. In spite of the observed general patterns of differences in place of death across the two groups of patients in each country, there were some additional cross-border differences. Home deaths were generally high for all decedents in Mexico (55.4–57.1%) and Italy (41.6–44.2%), whereas hospital deaths were high in France (60.2–77.4%) and South Korea (78.9–86.5%). The trend in France might be a result of the continued dominance of hospital-centered care and the insufficient training of oncologists and pulmonologists in palliative care. This might be understood in the light of different cultures of caregiving as well as of the surrounding medical culture. In those countries where hospice was recorded as a category, lung cancer patients were found to die there in far greater proportions than COPD patients. However, this was not the case in the USA and it might reflect how countries differ in placing the long-term focus of their palliative care services on cancer as opposed to other illnesses eligible for palliative care.^7^ More wider structural country-specific factors, such as insurance and reimbursement systems and regulations might, however, also play a role.

### Implications for future research, policy, and practice

In order to create equal opportunities for dying at home and for access to palliative care for COPD sufferers, a “palliative care” culture for COPD is needed as well as the awareness (both in caregivers and in patients) that early discussions about end-of-life care preferences might result in better care according to the patients’ wishes and less hospitalizations and more home deaths,^10^ although the trajectory and the death of COPD sufferers may be more difficult to accurately predict.

## Conclusions

Our study found in almost all countries that COPD sufferers as compared with lung cancer sufferers are less likely to die at home and more likely to die in a hospital or a nursing home. In the four countries that record palliative care institutions as a place of death, we found lung cancer sufferers to be much more likely to die there than COPD patients.

## Materials and methods

### Study design and data

This study is part of the International Place of Death (IPoD) study, which is a study of population-level death certificate data. An open call was launched by the principal investigators and candidate partners negotiated a full year’s death certificate data for inclusion. An exploration by all candidate partners revealed the most recent available year in all targeted countries was or would be 2008, which was chosen as the reference year. Exceptions were the USA (2007) and Spain (no data were recorded prior to 2010). Fourteen out of the 27 candidate countries obtained permission for data use and their data were integrated into an international database. The principal investigators pooled all data guaranteeing uniform coding throughout the database.

Death certification was executed in similar ways in the 14 countries: a physician or a qualified person such as a nurse completes the part of the death certificate indicating cause of death, time, and place of death^37^ along with a limited range of demographic information (e.g., sex) for the deceased. In some countries another part of the death certificate, containing more socio-demographic information about the deceased, is completed by a civil servant. All information is then processed by trained coders, following strict coding protocols, with the necessary quality checks. The death certificate data were linked across a number of countries with similar population databases such as the Census Data to include more socio-demographic information about the decedents in the database. For this study we used the death certificate data of all 14 countries included in the IPoD study: Belgium, France, Italy, Spain (Andalusia), the Netherlands, The Czech Republic, Hungary, England, Wales, New Zealand, the USA, Canada, Mexico, and Korea. More details on the database and methods can also be found in other publications based on the IPoD database.^38–40^

### Data

We selected cases where lung cancer (ICD-10 codes C33-C34) or COPD (ICD-10 codes J40-44, J47) was an underlying cause of death. The outcome for our study was the place of death as recorded in the death certificate. The available categories of place of death were: hospital, home, nursing home/residential long-term care, hospice, or others. In Hungary, the death certificate only contains two categories of place of death, hospital and others, whereas hospice (e.g., palliative care institution) was only available as a category in England, Wales, New Zealand, Canada, and the USA.

### Statistical analysis

Descriptive statistics were used to examine differences in the place of death between patients dying from lung cancer and those from COPD. Crude ratios (the percentages of lung cancer deaths divided by the percentages of COPD deaths) were calculated to compare the differences in place of death between the two disease groups.

Multivariable binary logistic regression models were constructed to determine the odds ratios of dying at home (comparing home vs. all other places and comparing home vs. hospital), in hospital (vs. all other places), in nursing home (vs. hospital), and in PC institutions (vs. all other places). All analyses used lung cancer as the reference group. Independent variables used in the multivariable analyses included demographics and health-care resources. Demographic factors included age (categories 0–17, 18–64, 65–85, 85 or above), sex, and marital status (unmarried, married, widowed, or divorced). Relevant confounders and covariates in the models were entered using a forward stepwise selection method with *P* < 0.05 set as an entry criterion.

All statistical analyses were conducted using IBM-SPSS Statistics version 20 (SPSS Inc., Chicago, IL, 2010). For all analyses, significance was set at *P* < .05 (two-tailed).

## Electronic supplementary material

Supplementary Information
