# Supplementary material for: Differences in place of death between lung cancer and COPD patients: a 14-country study using death certificate data
Source: NPJ Prim Care Respir Med. 2017 Mar 3;27:14. doi: 10.1038/s41533-017-0017-y (PMC5434782; doi:10.1038/s41533-017-0017-y)
Supplement: Supplementary file 1 — Supplementary Information [file 41533_2017_17_MOESM1_ESM.docx]

Supplemental Table S1: Place of death for all deaths in 14 countries

| Country |  | Hospital | Home | Nursing home | Hospice setting | other |
| --- | --- | --- | --- | --- | --- | --- |
| France | 541,135 | 57.5% | 26.7% | 10.9% | /^*^ | 4.8% |
| Italy | 578,192 | 47.0% | 41.8% | 6.4% | /^*^ | 4.8% |
| Spain (Andalusia) | 57,380 | 57.7% | 33.8% | 7.9% | /^*^ | 0.5% |
| Belgium | 102,924 | 50.5% | 23.9% | 23.2% | /^*^ | 2.4% |
| The Netherlands | 135,136 | 31.3% | 27.3% | 34.1% | /^*^ | 7.3%**^＋^** |
| Czech Republic | 101,804 | 57.4% | 21.6% | 17.7% | /^*^ | 3.4% |
| Hungary | 130,027 | 61.7% | /^*^ | /^*^ | /^*^ | 38.3% |
| England | 475,763 | 55.3% | 19.9% | 17.5% | 5.0% | 2.2% |
| Wales | 32,066 | 62.6% | 19.8% | 12.3% | 2.8% | 2.5% |
| New Zealand | 29,312 | 34.1% | 22.2% | 31.0% | 6.3% | 6.4% |
| Canada | 182,134 | 60.7% | 14.9% | 18.3% | /^*^ | 6.1% |
| United States of America | 2,428,343 | 43.9% | 25.4% | 21.7% | 2.7% | 6.2% |
| Mexico | 528,093 | 46.9% | 44.6% | /^*^ | /^*^ | 8.5% |
| Korea | 247,757 | 72.7% | 22.4% | 1.6% | /^*^ | 3.4% |

**^*^**Category not presented on death certificate. In Hungary, the death certificate registry only coded hospital or others as the place of death and nursing home does not exist as a separate health service in Mexico.

**^＋^**This includes the category ‘other institutions’ in the Netherlands (3.5% of all deaths) which often concerns hospices
